# Bis[μ-1,3-bis­(1*H*-imidazol-1-yl)propane-κ^2^ *N* ^3^:*N* ^3′^]bis­(di­chlorido­zinc) dihydrate

**DOI:** 10.1107/S1600536814008162

**Published:** 2014-04-16

**Authors:** Xiao-Juan Wang, Yun-Long Feng

**Affiliations:** a College of Chemistry and Life Science, Zhejiang Normal University, Jinhua, Zhejiang 321004, People’s Republic of China

## Abstract

The title hydrated complex, [Zn_2_Cl_4_(C_9_H_12_N_4_)_2_]·2H_2_O, is a discrete dinuclear zinc complex with 1,3-bis­(1*H*-imidazol-1-yl)propane as the bridging ligand. The complex mol­ecule lies about a crystallographic inversion centre. The Zn^II^ atom exhibits a distorted tetra­hedral coordination geometry defined by two imidazole N atoms and two Cl atoms. O—H⋯Cl hydrogen bonding between the lattice water mol­ecules and the terminal Cl atoms of the mol­ecule lead to a two-dimensional structure extending parallel to (100).

## Related literature   

For related structures containing the 1,3-bis­(imidazol)propane ligand, see: Ma *et al.* (2012 ▶); Kan *et al.* (2012 ▶); Jiang *et al.* (2011 ▶); Shen & Lin (2012 ▶).
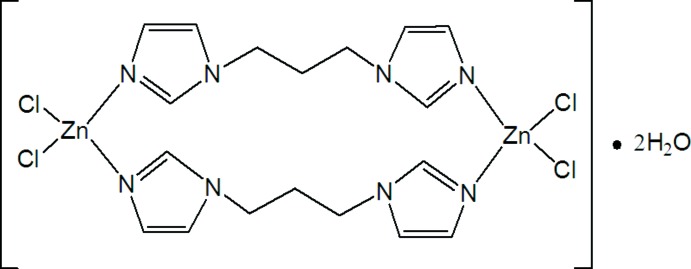


## Experimental   

### 

#### Crystal data  

[Zn_2_Cl_4_(C_9_H_12_N_4_)_2_]·2H_2_O
*M* *_r_* = 661.02
Monoclinic, 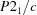

*a* = 10.1378 (4) Å
*b* = 9.7173 (4) Å
*c* = 13.8801 (6) Å
β = 93.704 (2)°
*V* = 1364.50 (10) Å^3^
*Z* = 2
Mo *K*α radiation
μ = 2.18 mm^−1^
*T* = 296 K
0.25 × 0.18 × 0.12 mm

#### Data collection  

Bruker APEXII CCD diffractometer
Absorption correction: multi-scan (*SADABS*; Bruker, 2006 ▶) *T*_min_ = 0.631, *T*_max_ = 0.770
21184 measured reflections
3162 independent reflections
2473 reflections with *I* > 2σ(*I*)
*R*_int_ = 0.033

#### Refinement  

*R*[*F* ^2^ > 2σ(*F* ^2^)] = 0.030
*wR*(*F* ^2^) = 0.087
*S* = 1.04
3162 reflections
154 parameters
H-atom parameters constrained
Δρ_max_ = 0.55 e Å^−3^
Δρ_min_ = −0.23 e Å^−3^

### 

Data collection: *APEX2* (Bruker, 2006 ▶); cell refinement: *SAINT* (Bruker, 2006 ▶); data reduction: *SAINT*; program(s) used to solve structure: *SHELXS97* (Sheldrick, 2008 ▶); program(s) used to refine structure: *SHELXL97* (Sheldrick, 2008 ▶); molecular graphics: *DIAMOND* (Brandenburg, 1999 ▶); software used to prepare material for publication: *publCIF* (Westrip, 2010 ▶).

## Supplementary Material

Crystal structure: contains datablock(s) I. DOI: 10.1107/S1600536814008162/wm5017sup1.cif

Structure factors: contains datablock(s) I. DOI: 10.1107/S1600536814008162/wm5017Isup2.hkl

Supporting information file. DOI: 10.1107/S1600536814008162/wm5017Isup3.mol

CCDC reference: 996742

Additional supporting information:  crystallographic information; 3D view; checkCIF report

## Figures and Tables

**Table 1:** Hydrogen-bond geometry (Å, °)

*D*—H⋯*A*	*D*—H	H⋯*A*	*D*⋯*A*	*D*—H⋯*A*
O1*W*—H1*WA*⋯Cl1	0.85	2.47	3.282 (3)	160
O1*W*—H1*WB*⋯Cl2^i^	0.85	2.76	3.473 (4)	143

Symmetry code: (i) 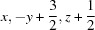.

**Fig. 1.**
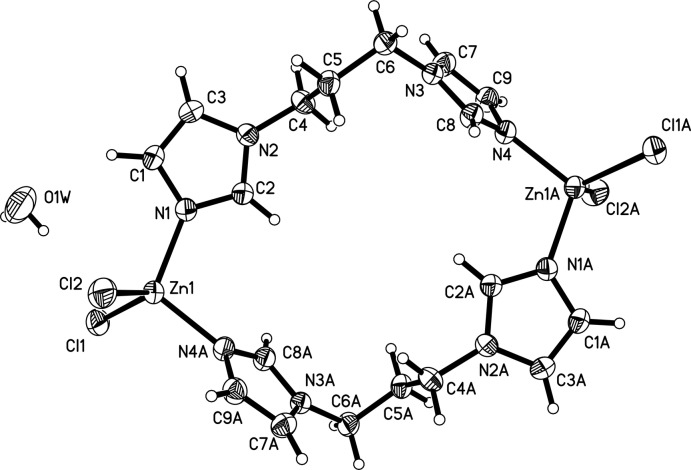
Perspective view of the molecular entities of (I), showing the atom-numbering scheme. Displacement ellipsoids are drawn at the 30% probability level. [Symmetry code: (A) -x + 1,-y + 1,-z + 1.]

**Fig. 2.**
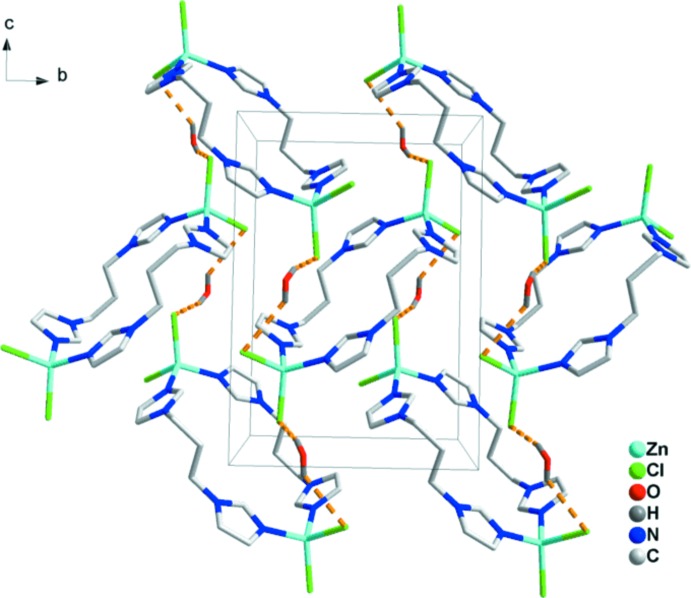
The layer structure of (I) viewed along [100]. Dashed lines indicate O—H···Cl hydrogen bonds.

## supplementary crystallographic information

### 1. Comment

In the past few years, complexes based on the 1,3-bis(imidazol)propane (1,3-bip) ligand have been reported, such as [Mn_4_(tbip)_4_(1,3-bip)]_n_^.^2nH_2_O (H_2_tbip = (5-*tert*-butyl isophthalic acid) (Ma *et al.*, 2012), [Cd(H*L*)(1,3-bip)]_n_^.^5nH_2_O (H_3_*L* = 5-(2-carboxybenzyloxy)isophthalic acid) (Kan *et al.*, 2012), [Zn(*L*)(1,3-bip)]_n_ (H_2_*L* = 5-methylisophthalic acid) (Jiang *et al.*, 2011), [Cd(1,3-bip)Cl_2_]_n_ (Shen *et al.*, 2012). In order to extend our knowledge in this field, we report here the syntheses and structure of a new complex, [ZnCl_2_(C_9_H_12_N_4_]_2_^.^2H_2_O, (I).

The asymmetric unit of (I) consists of one Zn^2+^ ion, one 1,3-bip ligand, two Cl^-^ ions, and one lattice water molecules. A perspective view of the molecular entities of complex (I) is presented in Fig. 1. The complex contains centrosymmetric dimers with bridging 1,3-bip ligands. The Zn(II) atom is four-coordinated in a distorted tetrahedral coordination. O—H···Cl hydrogen bonds between the lattice water molecules and Cl atoms lead to a layered structure extending parallel to (100) (Fig. 2).

### 2. Experimental

A mixture of 1,3-bis(imidazol)propane (0.088 g, 0.5 mmol), ZnCl_2_ (0.204 g, 1.5 mmol), and Na_2_CO_3_ (0.060 g, 0.5 mmol) in H_2_O (16 ml)/C_2_H_5_OH (2 ml) was placed in a 25 ml Teflon-lined stainless steel vessel and heated at 433 K for 72 h, then cooled to room temperature over a period of 24 h. Colourless crystals suitable for X-ray analysis were obtained.

### 3. Refinement

The carbon-bound H-atoms were positioned geometrically and included in the refinement using a riding model [aromatic C—H 0.93 Å and aliphatic C—H 0.97 Å, *U*_iso_(H) = 1.2*U*_eq_(C)]. The oxygen-bound H-atoms were located in a difference Fourier map and were refined with the O—H distance restraint of 0.85 Å [*U*_iso_(H) = 1.2*U*_eq_(O)].

### Crystal data

**Table:**

[Zn_2_Cl_4_(C_9_H_12_N_4_)_2_]·2H_2_O	*F*(000) = 672
*M**_r_* = 661.02	*D*_x_ = 1.609 Mg m^−^^3^
Monoclinic, *P*2_1_/*c*	Mo *K*α radiation, λ = 0.71073 Å
Hall symbol: -P 2ybc	Cell parameters from 6173 reflections
*a* = 10.1378 (4) Å	θ = 2.0–27.6°
*b* = 9.7173 (4) Å	µ = 2.18 mm^−^^1^
*c* = 13.8801 (6) Å	*T* = 296 K
β = 93.704 (2)°	Block, colourless
*V* = 1364.50 (10) Å^3^	0.25 × 0.18 × 0.12 mm
*Z* = 2	

### Data collection

**Table:**

Bruker APEXII CCD diffractometer	3162 independent reflections
Radiation source: fine-focus sealed tube	2473 reflections with *I* > 2σ(*I*)
Graphite monochromator	*R*_int_ = 0.033
ω and φ–scans	θ_max_ = 27.6°, θ_min_ = 2.0°
Absorption correction: multi-scan (*SADABS*; Bruker, 2006)	*h* = −13→13
*T*_min_ = 0.631, *T*_max_ = 0.770	*k* = −11→12
21184 measured reflections	*l* = −18→18

### Refinement

**Table:**

Refinement on *F*^2^	Primary atom site location: structure-invariant direct methods
Least-squares matrix: full	Secondary atom site location: difference Fourier map
*R*[*F*^2^ > 2σ(*F*^2^)] = 0.030	Hydrogen site location: inferred from neighbouring sites
*wR*(*F*^2^) = 0.087	H-atom parameters constrained
*S* = 1.04	*w* = 1/[σ^2^(*F*_o_^2^) + (0.0432*P*)^2^ + 0.5084*P*] where *P* = (*F*_o_^2^ + 2*F*_c_^2^)/3
3162 reflections	(Δ/σ)_max_ = 0.001
154 parameters	Δρ_max_ = 0.55 e Å^−^^3^
0 restraints	Δρ_min_ = −0.23 e Å^−^^3^

### Special details

**Table:**

Geometry. All e.s.d.'s (except the e.s.d. in the dihedral angle between two l.s. planes) are estimated using the full covariance matrix. The cell e.s.d.'s are taken into account individually in the estimation of e.s.d.'s in distances, angles and torsion angles; correlations between e.s.d.'s in cell parameters are only used when they are defined by crystal symmetry. An approximate (isotropic) treatment of cell e.s.d.'s is used for estimating e.s.d.'s involving l.s. planes.
Refinement. Refinement of *F*^2^ against ALL reflections. The weighted *R*-factor *wR* and goodness of fit *S* are based on *F*^2^, conventional *R*-factors *R* are based on *F*, with *F* set to zero for negative *F*^2^. The threshold expression of *F*^2^ > σ(*F*^2^) is used only for calculating *R*-factors(gt) *etc*. and is not relevant to the choice of reflections for refinement. *R*-factors based on *F*^2^ are statistically about twice as large as those based on *F*, and *R*- factors based on ALL data will be even larger.

### Fractional atomic coordinates and isotropic or equivalent isotropic displacement parameters (Å^2^)

**Table:**

	*x*	*y*	*z*	*U*_iso_*/*U*_eq_	
Zn1	0.34226 (3)	0.80572 (3)	0.74784 (2)	0.04641 (11)	
Cl1	0.39773 (7)	0.82340 (8)	0.90676 (5)	0.0634 (2)	
Cl2	0.19959 (7)	0.97104 (7)	0.69087 (6)	0.0684 (2)	
O1W	0.1166 (3)	0.7064 (4)	0.9784 (2)	0.1265 (12)	
H1WA	0.1788	0.7355	0.9456	0.152*	
H1WB	0.1429	0.7043	1.0377	0.152*	
N1	0.25814 (18)	0.6260 (2)	0.70857 (13)	0.0443 (4)	
N2	0.21115 (19)	0.43655 (19)	0.62798 (13)	0.0430 (4)	
N3	0.29331 (19)	0.1944 (2)	0.37271 (14)	0.0452 (4)	
N4	0.49316 (19)	0.1728 (2)	0.32351 (14)	0.0458 (5)	
C1	0.1371 (2)	0.5783 (3)	0.73318 (17)	0.0474 (6)	
H1A	0.0841	0.6199	0.7771	0.057*	
C2	0.2989 (2)	0.5376 (2)	0.64438 (16)	0.0449 (5)	
H2A	0.3782	0.5449	0.6146	0.054*	
C3	0.1072 (2)	0.4622 (3)	0.68377 (17)	0.0481 (6)	
H3A	0.0310	0.4096	0.6869	0.058*	
C4	0.2215 (3)	0.3244 (2)	0.55803 (18)	0.0505 (6)	
H4A	0.1746	0.2443	0.5797	0.061*	
H4B	0.3137	0.2993	0.5542	0.061*	
C5	0.1646 (2)	0.3666 (3)	0.45951 (16)	0.0489 (5)	
H5A	0.2165	0.4420	0.4363	0.059*	
H5B	0.0752	0.3999	0.4651	0.059*	
C6	0.1616 (2)	0.2514 (3)	0.3861 (2)	0.0555 (6)	
H6A	0.1046	0.1785	0.4068	0.067*	
H6B	0.1241	0.2858	0.3247	0.067*	
C7	0.3435 (3)	0.0752 (3)	0.41050 (19)	0.0571 (6)	
H7A	0.3011	0.0141	0.4498	0.069*	
C8	0.3855 (2)	0.2498 (3)	0.32038 (17)	0.0489 (6)	
H8A	0.3753	0.3318	0.2862	0.059*	
C9	0.4668 (3)	0.0622 (3)	0.38023 (18)	0.0533 (6)	
H9A	0.5245	−0.0101	0.3955	0.064*	

### Atomic displacement parameters (Å^2^)

**Table:**

	*U*^11^	*U*^22^	*U*^33^	*U*^12^	*U*^13^	*U*^23^
Zn1	0.04298 (17)	0.04543 (18)	0.05161 (18)	0.00076 (12)	0.00928 (12)	−0.00741 (12)
Cl1	0.0657 (4)	0.0750 (5)	0.0500 (4)	−0.0032 (3)	0.0081 (3)	−0.0131 (3)
Cl2	0.0546 (4)	0.0569 (4)	0.0943 (5)	0.0137 (3)	0.0087 (4)	0.0021 (4)
O1W	0.0764 (17)	0.190 (4)	0.116 (2)	0.0006 (18)	0.0274 (16)	−0.022 (2)
N1	0.0451 (10)	0.0451 (11)	0.0431 (10)	0.0013 (9)	0.0051 (8)	−0.0024 (8)
N2	0.0450 (10)	0.0398 (10)	0.0437 (10)	0.0030 (8)	0.0000 (8)	−0.0004 (8)
N3	0.0391 (10)	0.0488 (12)	0.0480 (11)	−0.0025 (8)	0.0062 (8)	−0.0095 (9)
N4	0.0453 (11)	0.0478 (12)	0.0451 (11)	0.0026 (9)	0.0088 (8)	−0.0006 (8)
C1	0.0415 (12)	0.0553 (15)	0.0462 (12)	0.0047 (11)	0.0082 (10)	0.0008 (10)
C2	0.0439 (12)	0.0435 (13)	0.0477 (12)	−0.0006 (10)	0.0070 (10)	−0.0029 (10)
C3	0.0403 (12)	0.0522 (15)	0.0518 (13)	−0.0034 (10)	0.0029 (10)	0.0065 (11)
C4	0.0585 (15)	0.0388 (13)	0.0536 (14)	0.0029 (11)	−0.0008 (11)	−0.0051 (10)
C5	0.0466 (13)	0.0520 (14)	0.0486 (13)	0.0060 (11)	0.0062 (10)	−0.0028 (11)
C6	0.0366 (12)	0.0696 (17)	0.0604 (15)	0.0003 (12)	0.0040 (11)	−0.0170 (13)
C7	0.0603 (16)	0.0513 (15)	0.0614 (15)	−0.0057 (12)	0.0171 (12)	0.0044 (12)
C8	0.0467 (13)	0.0493 (14)	0.0514 (14)	0.0031 (11)	0.0094 (11)	0.0013 (11)
C9	0.0574 (15)	0.0461 (14)	0.0570 (14)	0.0063 (11)	0.0088 (12)	0.0011 (11)

### Geometric parameters (Å, º)

**Table:**

Zn1—N1	2.0038 (19)	C1—C3	1.345 (3)
Zn1—N4^i^	2.0053 (19)	C1—H1A	0.9300
Zn1—Cl1	2.2476 (7)	C2—H2A	0.9300
Zn1—Cl2	2.2694 (7)	C3—H3A	0.9300
O1W—H1WA	0.8500	C4—C5	1.507 (3)
O1W—H1WB	0.8500	C4—H4A	0.9700
N1—C2	1.323 (3)	C4—H4B	0.9700
N1—C1	1.375 (3)	C5—C6	1.513 (3)
N2—C2	1.334 (3)	C5—H5A	0.9700
N2—C3	1.370 (3)	C5—H5B	0.9700
N2—C4	1.468 (3)	C6—H6A	0.9700
N3—C8	1.333 (3)	C6—H6B	0.9700
N3—C7	1.357 (3)	C7—C9	1.350 (4)
N3—C6	1.468 (3)	C7—H7A	0.9300
N4—C8	1.322 (3)	C8—H8A	0.9300
N4—C9	1.369 (3)	C9—H9A	0.9300
N4—Zn1^i^	2.0053 (19)		
			
N1—Zn1—N4^i^	108.03 (8)	N2—C4—C5	111.01 (19)
N1—Zn1—Cl1	114.12 (6)	N2—C4—H4A	109.4
N4^i^—Zn1—Cl1	108.26 (6)	C5—C4—H4A	109.4
N1—Zn1—Cl2	105.77 (6)	N2—C4—H4B	109.4
N4^i^—Zn1—Cl2	106.63 (6)	C5—C4—H4B	109.4
Cl1—Zn1—Cl2	113.66 (3)	H4A—C4—H4B	108.0
H1WA—O1W—H1WB	109.3	C4—C5—C6	113.6 (2)
C2—N1—C1	105.7 (2)	C4—C5—H5A	108.8
C2—N1—Zn1	127.09 (16)	C6—C5—H5A	108.8
C1—N1—Zn1	126.64 (16)	C4—C5—H5B	108.8
C2—N2—C3	107.35 (19)	C6—C5—H5B	108.8
C2—N2—C4	125.7 (2)	H5A—C5—H5B	107.7
C3—N2—C4	126.8 (2)	N3—C6—C5	112.65 (19)
C8—N3—C7	107.2 (2)	N3—C6—H6A	109.1
C8—N3—C6	126.3 (2)	C5—C6—H6A	109.1
C7—N3—C6	126.5 (2)	N3—C6—H6B	109.1
C8—N4—C9	105.8 (2)	C5—C6—H6B	109.1
C8—N4—Zn1^i^	129.55 (17)	H6A—C6—H6B	107.8
C9—N4—Zn1^i^	124.41 (16)	C9—C7—N3	106.9 (2)
C3—C1—N1	109.3 (2)	C9—C7—H7A	126.5
C3—C1—H1A	125.3	N3—C7—H7A	126.5
N1—C1—H1A	125.3	N4—C8—N3	111.1 (2)
N1—C2—N2	111.0 (2)	N4—C8—H8A	124.4
N1—C2—H2A	124.5	N3—C8—H8A	124.4
N2—C2—H2A	124.5	C7—C9—N4	108.9 (2)
C1—C3—N2	106.6 (2)	C7—C9—H9A	125.5
C1—C3—H3A	126.7	N4—C9—H9A	125.5
N2—C3—H3A	126.7		
			
N4^i^—Zn1—N1—C2	−2.4 (2)	C2—N2—C4—C5	−86.8 (3)
Cl1—Zn1—N1—C2	−122.86 (18)	C3—N2—C4—C5	88.8 (3)
Cl2—Zn1—N1—C2	111.46 (19)	N2—C4—C5—C6	−175.0 (2)
N4^i^—Zn1—N1—C1	−172.98 (18)	C8—N3—C6—C5	−78.9 (3)
Cl1—Zn1—N1—C1	66.57 (19)	C7—N3—C6—C5	101.8 (3)
Cl2—Zn1—N1—C1	−59.12 (19)	C4—C5—C6—N3	−58.6 (3)
C2—N1—C1—C3	0.2 (3)	C8—N3—C7—C9	0.5 (3)
Zn1—N1—C1—C3	172.38 (16)	C6—N3—C7—C9	180.0 (2)
C1—N1—C2—N2	−0.6 (3)	C9—N4—C8—N3	0.6 (3)
Zn1—N1—C2—N2	−172.77 (14)	Zn1^i^—N4—C8—N3	174.77 (15)
C3—N2—C2—N1	0.8 (3)	C7—N3—C8—N4	−0.7 (3)
C4—N2—C2—N1	177.2 (2)	C6—N3—C8—N4	179.8 (2)
N1—C1—C3—N2	0.3 (3)	N3—C7—C9—N4	−0.2 (3)
C2—N2—C3—C1	−0.7 (2)	C8—N4—C9—C7	−0.2 (3)
C4—N2—C3—C1	−177.0 (2)	Zn1^i^—N4—C9—C7	−174.79 (17)

Symmetry code: (i) −*x*+1, −*y*+1, −*z*+1.

### Hydrogen-bond geometry (Å, º)

**Table:**

*D*—H···*A*	*D*—H	H···*A*	*D*···*A*	*D*—H···*A*
O1*W*—H1*WA*···Cl1	0.85	2.47	3.282 (3)	160
O1*W*—H1*WB*···Cl2^ii^	0.85	2.76	3.473 (4)	143

Symmetry code: (ii) *x*, −*y*+3/2, *z*+1/2.
